# Supplementary material for: FAM76B regulates NF-κB-mediated inflammatory pathway by influencing the translocation of hnRNPA2B1
Source: eLife. 2023 Aug 10;12:e85659. doi: 10.7554/eLife.85659 (PMC10446823; doi:10.7554/eLife.85659)
Supplement: Figure 1—source data 1. [file elife-85659-fig1-data1.zip › Figure 1-Labeled uncropped western blot images (source data 1-3)/Figure 1-source data 3.pdf]

Full unedited gel for Figure.1e

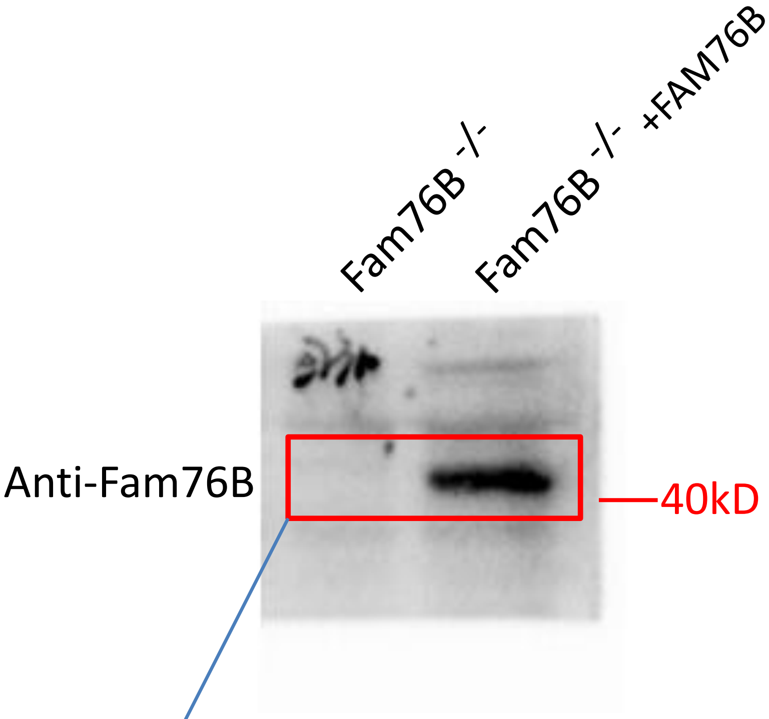

This lane corresponds to the band blotted with anti-FAM76B of Figure 1e in the cropped images within the manuscript.

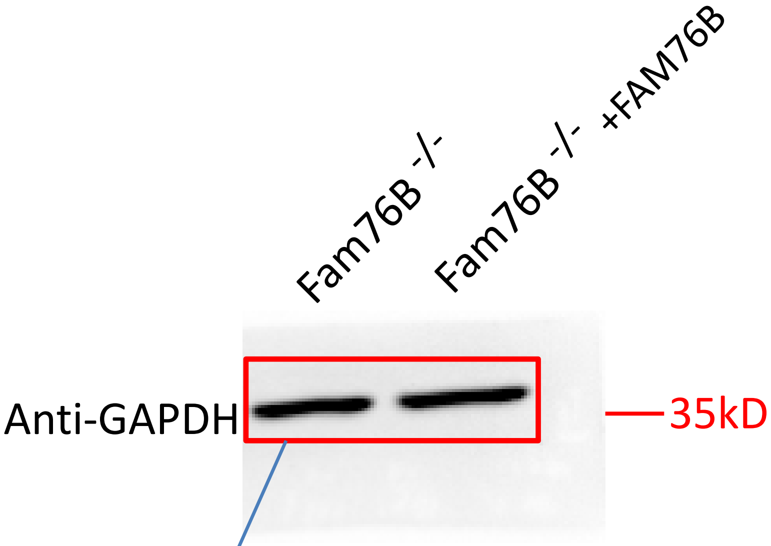

This lane corresponds to the band (anti-GAPDH) of Figure 1e in the cropped images within the manuscript.
