# Supplementary figures and images for: FAM76B regulates NF-κB-mediated inflammatory pathway by influencing the translocation of hnRNPA2B1
Source: eLife. 2023 Aug 10;12:e85659. doi: 10.7554/eLife.85659 (PMC10446823; doi:10.7554/eLife.85659)

## Full unedited gel for Figure.1a

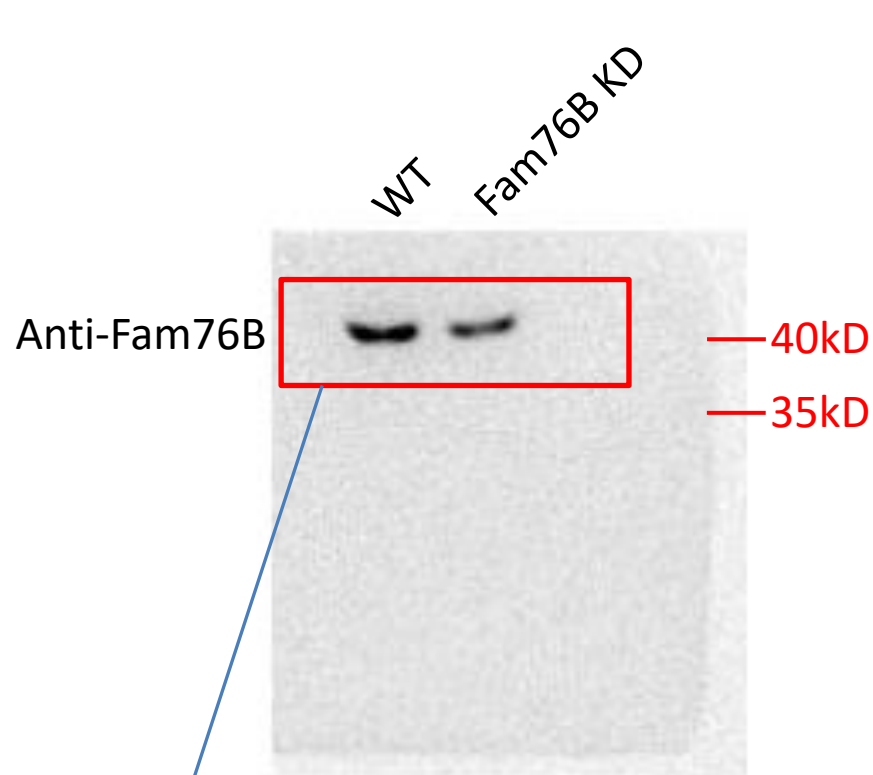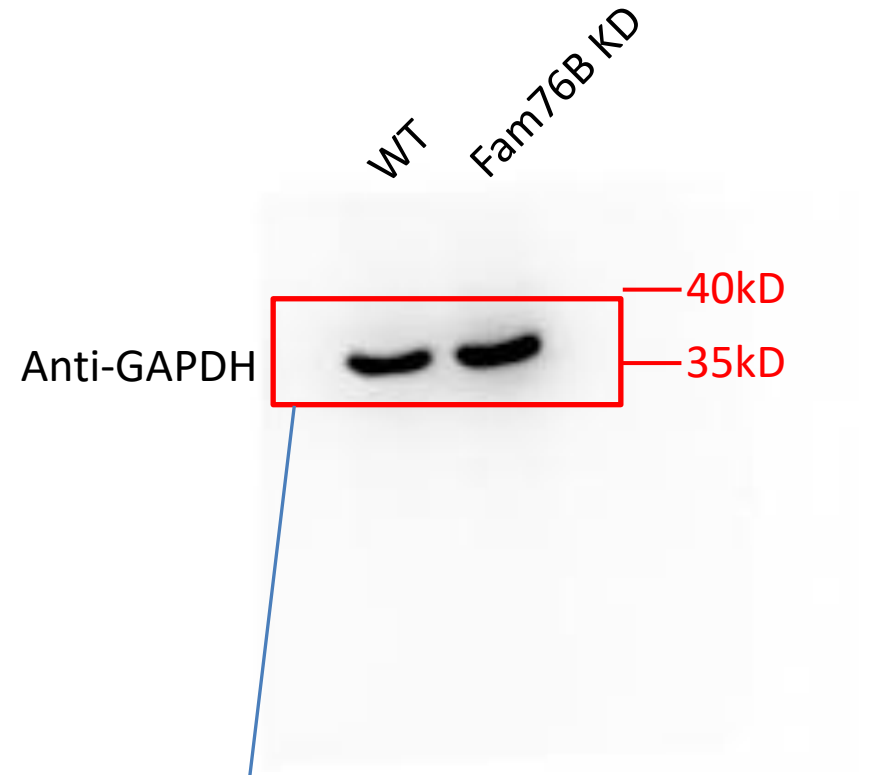

Supplement: Figure 1—source data 1. [file elife-85659-fig1-data1.zip › Figure 1-Labeled uncropped western blot images (source data 1-3)/Figure 1-source data 1.pdf]

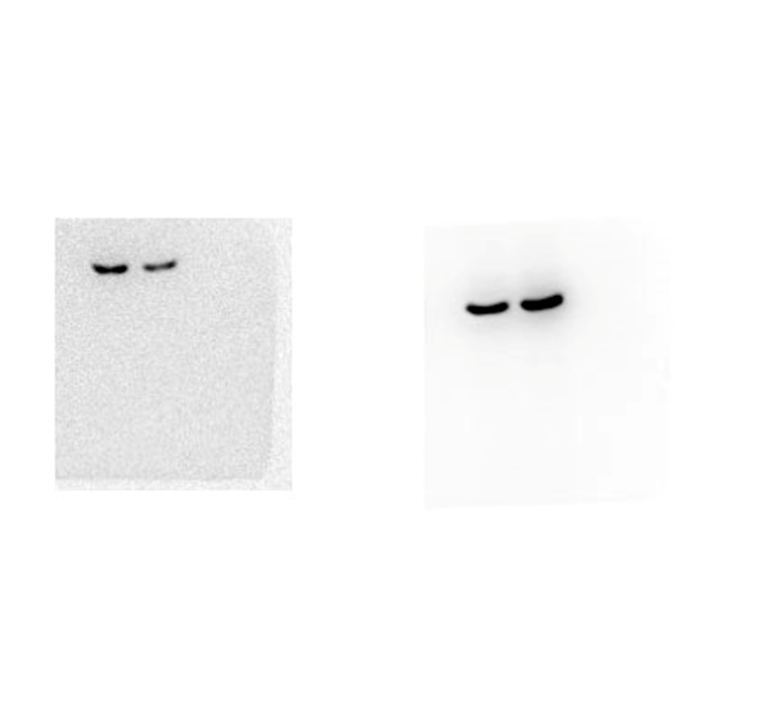

Supplement: Figure 1—source data 2. [file elife-85659-fig1-data2.zip › the raw unedited unlabelled versions of the gel images with tiff format ú¿Figure 1-source data 1-3)/Figure 1-source data 1.tif]

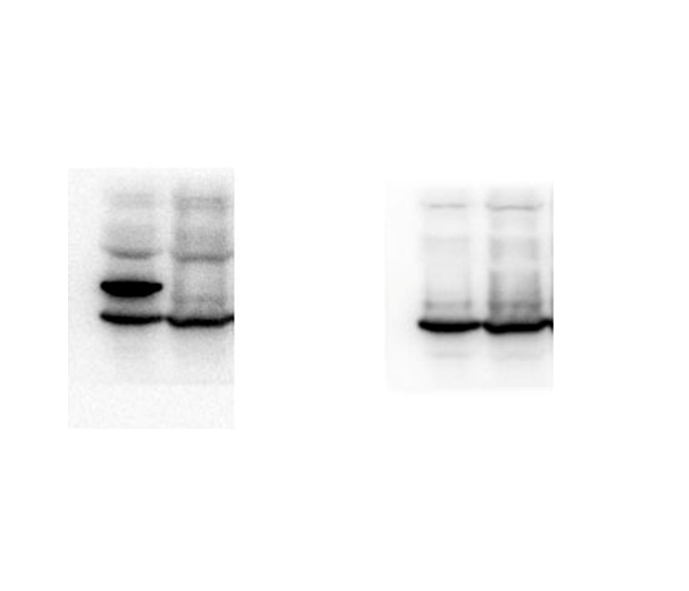

Supplement: Figure 1—source data 2. [file elife-85659-fig1-data2.zip › the raw unedited unlabelled versions of the gel images with tiff format ú¿Figure 1-source data 1-3)/Figure 1-source data 2.tif]

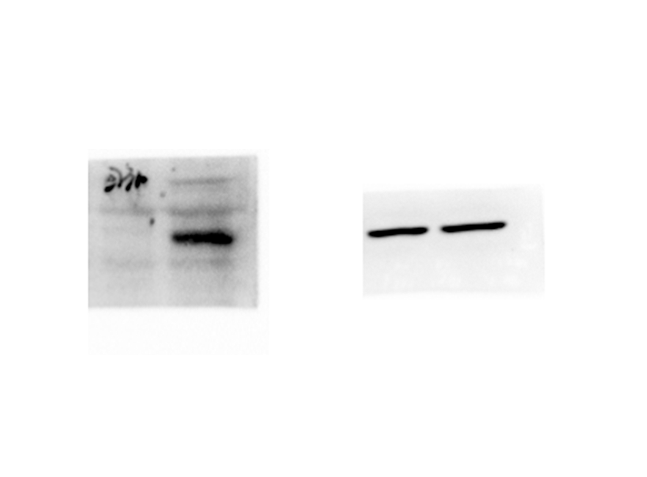

Supplement: Figure 1—source data 2. [file elife-85659-fig1-data2.zip › the raw unedited unlabelled versions of the gel images with tiff format ú¿Figure 1-source data 1-3)/Figure 1-source data 3.tif]

## Full unedited gel for Figure.3d

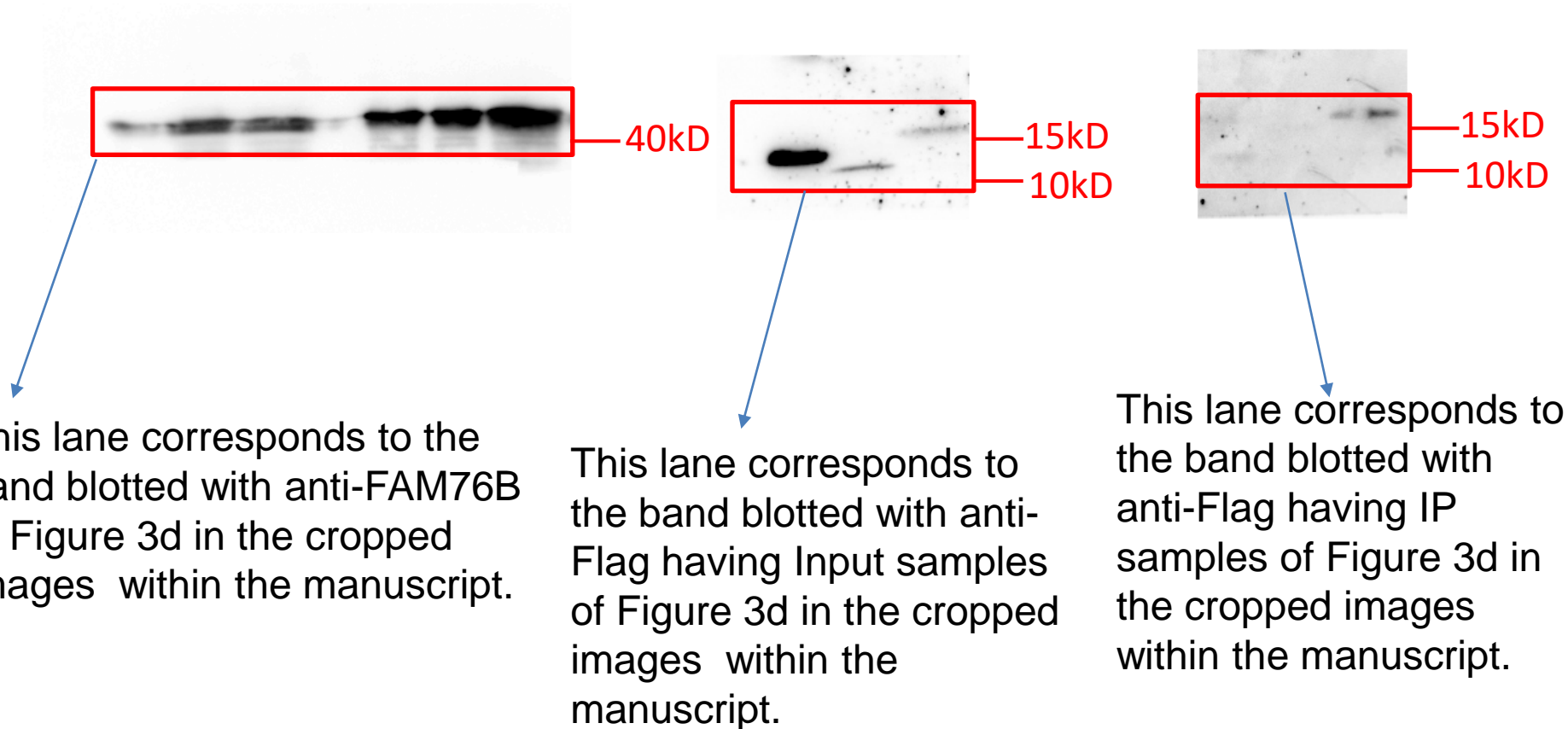

Supplement: Figure 3—source data 1. [file elife-85659-fig3-data1.zip › Figure 3-Labeled uncropped western blot images (source data 1-3)/Figure 3-Source data 2.pdf]

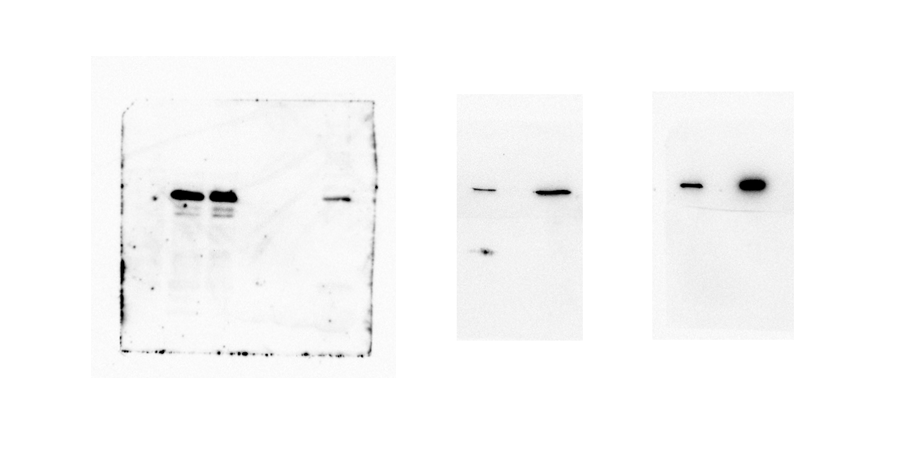

Supplement: Figure 3—source data 2. [file elife-85659-fig3-data2.zip › Figure 3-Raw western blot images with tiff format (source data 1-3)/Figure 3-source data 1.tif]

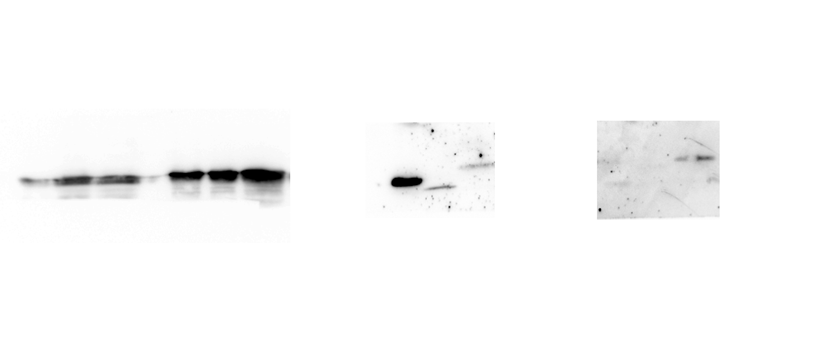

Supplement: Figure 3—source data 2. [file elife-85659-fig3-data2.zip › Figure 3-Raw western blot images with tiff format (source data 1-3)/Figure 3-source data 2.tif]

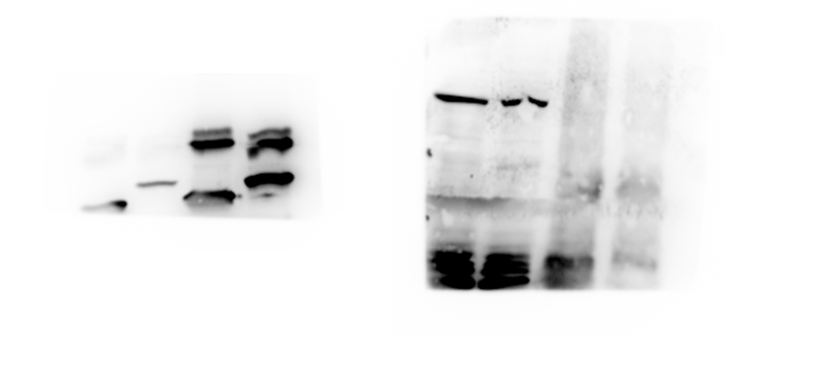

Supplement: Figure 3—source data 2. [file elife-85659-fig3-data2.zip › Figure 3-Raw western blot images with tiff format (source data 1-3)/Figure 3-source data 3.tif]

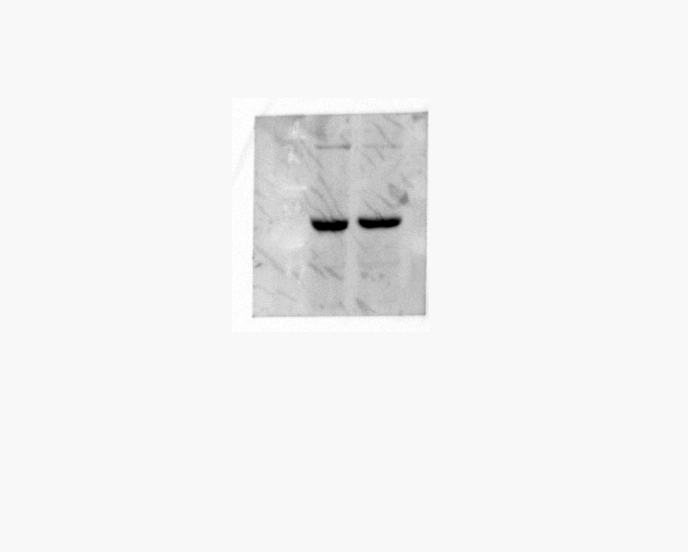

Supplement: Figure 4—source data 2. [file elife-85659-fig4-data2.zip › revised Figure 4 -raw unedited unlabelled versions of gel images with TIFF format/Source data 1 (Fig.4b)/Total β-actin.tif]

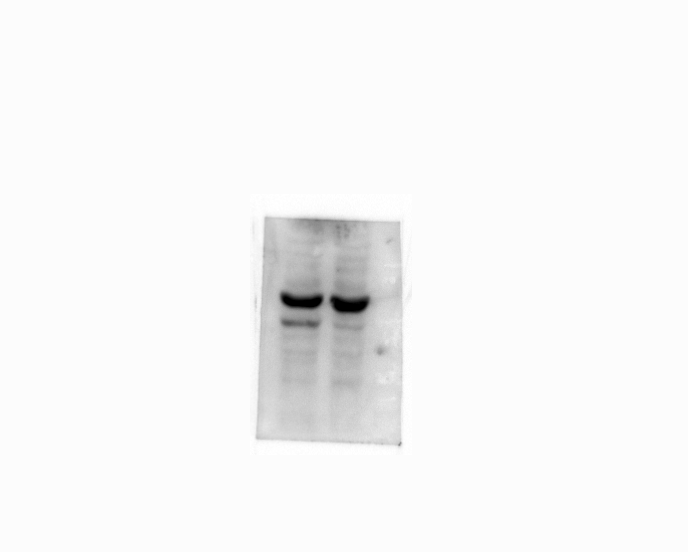

Supplement: Figure 4—source data 2. [file elife-85659-fig4-data2.zip › revised Figure 4 -raw unedited unlabelled versions of gel images with TIFF format/Source data 1 (Fig.4b)/cytoplasmic β-actin.tif]

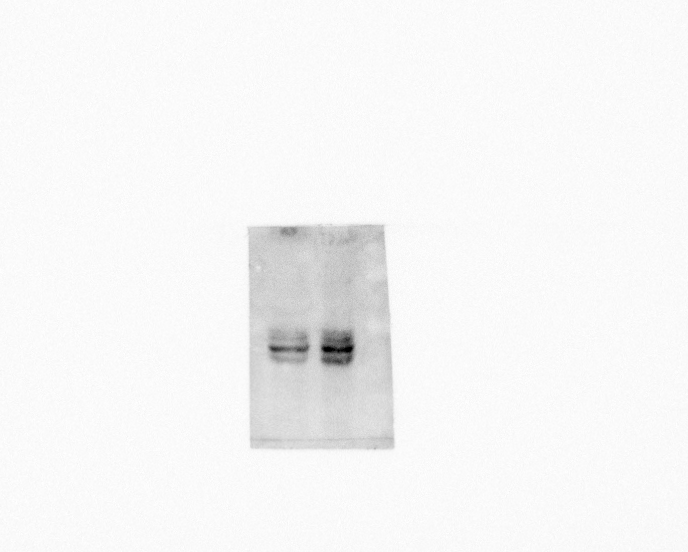

Supplement: Figure 4—source data 2. [file elife-85659-fig4-data2.zip › revised Figure 4 -raw unedited unlabelled versions of gel images with TIFF format/Source data 1 (Fig.4b)/cytoplasmic hnRNPA2B1.tif]

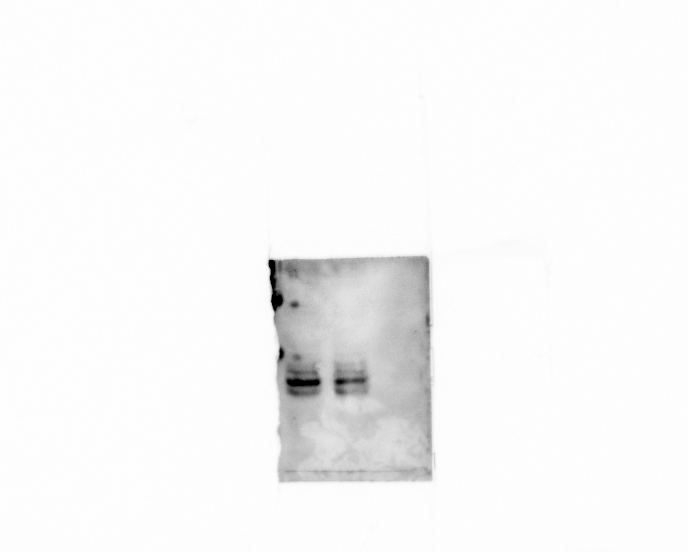

Supplement: Figure 4—source data 2. [file elife-85659-fig4-data2.zip › revised Figure 4 -raw unedited unlabelled versions of gel images with TIFF format/Source data 1 (Fig.4b)/nuclear hnRNPA2B1.tif]

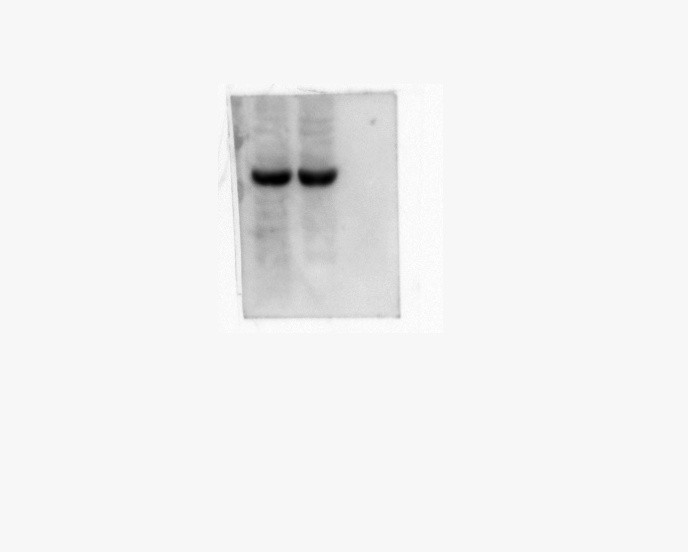

Supplement: Figure 4—source data 2. [file elife-85659-fig4-data2.zip › revised Figure 4 -raw unedited unlabelled versions of gel images with TIFF format/Source data 1 (Fig.4b)/nuclear laminB.tif]

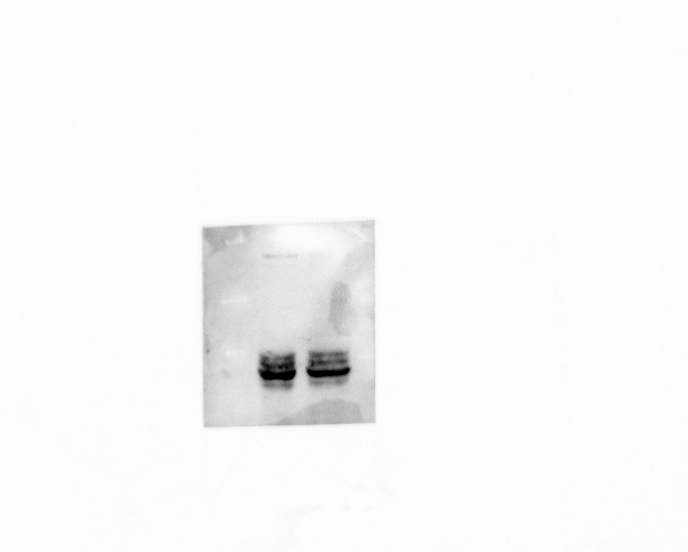

Supplement: Figure 4—source data 2. [file elife-85659-fig4-data2.zip › revised Figure 4 -raw unedited unlabelled versions of gel images with TIFF format/Source data 1 (Fig.4b)/total hnRNPA2B1.tif]

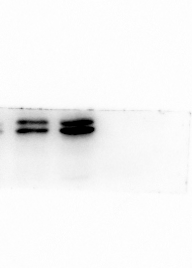

Supplement: Figure 4—source data 2. [file elife-85659-fig4-data2.zip › revised Figure 4 -raw unedited unlabelled versions of gel images with TIFF format/Source data 2 (Fig.4e)/cytoplasmic hnRNPA2B1.tif]

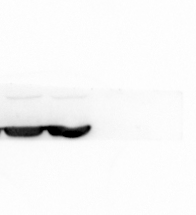

Supplement: Figure 4—source data 2. [file elife-85659-fig4-data2.zip › revised Figure 4 -raw unedited unlabelled versions of gel images with TIFF format/Source data 2 (Fig.4e)/cytoplasmic β-actin.tif]

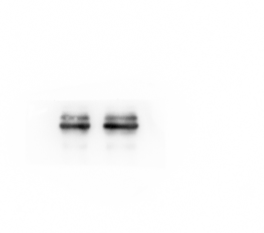

Supplement: Figure 4—source data 2. [file elife-85659-fig4-data2.zip › revised Figure 4 -raw unedited unlabelled versions of gel images with TIFF format/Source data 2 (Fig.4e)/nuclear hnRNPA2B1.tif]

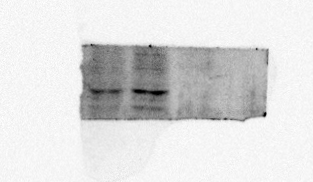

Supplement: Figure 4—source data 2. [file elife-85659-fig4-data2.zip › revised Figure 4 -raw unedited unlabelled versions of gel images with TIFF format/Source data 2 (Fig.4e)/nuclear laminB.tif]

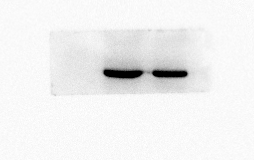

Supplement: Figure 4—source data 2. [file elife-85659-fig4-data2.zip › revised Figure 4 -raw unedited unlabelled versions of gel images with TIFF format/Source data 2 (Fig.4e)/total β-actin.tif]

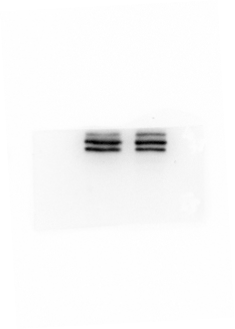

Supplement: Figure 4—source data 2. [file elife-85659-fig4-data2.zip › revised Figure 4 -raw unedited unlabelled versions of gel images with TIFF format/Source data 2 (Fig.4e)/total hnRNPA2B1.tif]

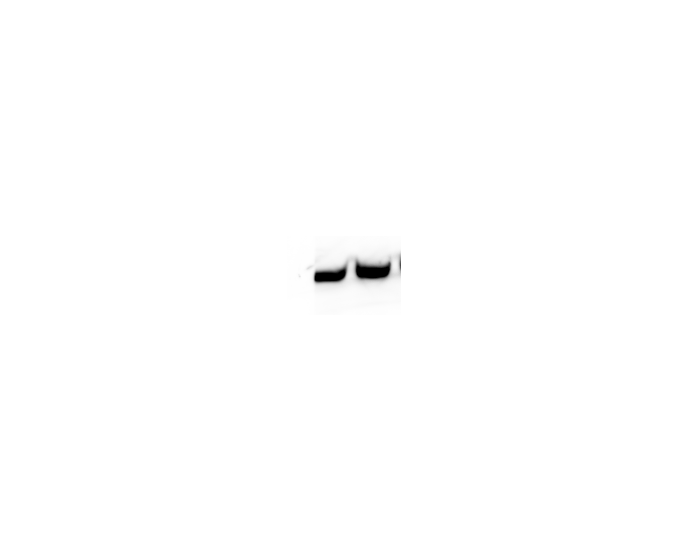

Supplement: Figure 4—source data 2. [file elife-85659-fig4-data2.zip › revised Figure 4 -raw unedited unlabelled versions of gel images with TIFF format/Source data 3 (Fig.4g)/GAPDH.tif]

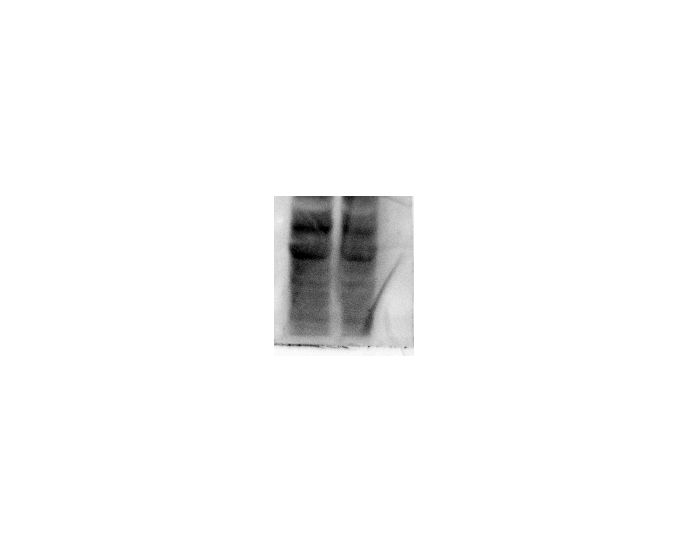

Supplement: Figure 4—source data 2. [file elife-85659-fig4-data2.zip › revised Figure 4 -raw unedited unlabelled versions of gel images with TIFF format/Source data 3 (Fig.4g)/IKBα.tif]

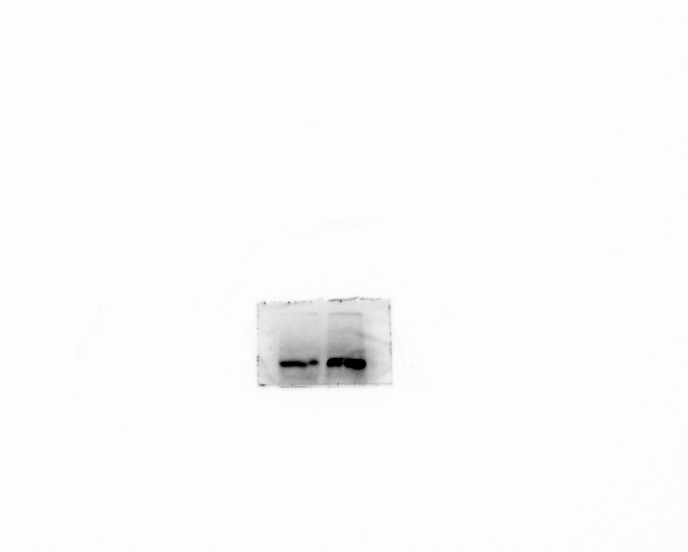

Supplement: Figure 4—source data 2. [file elife-85659-fig4-data2.zip › revised Figure 4 -raw unedited unlabelled versions of gel images with TIFF format/Source data 3 (Fig.4g)/IKK β.tif]

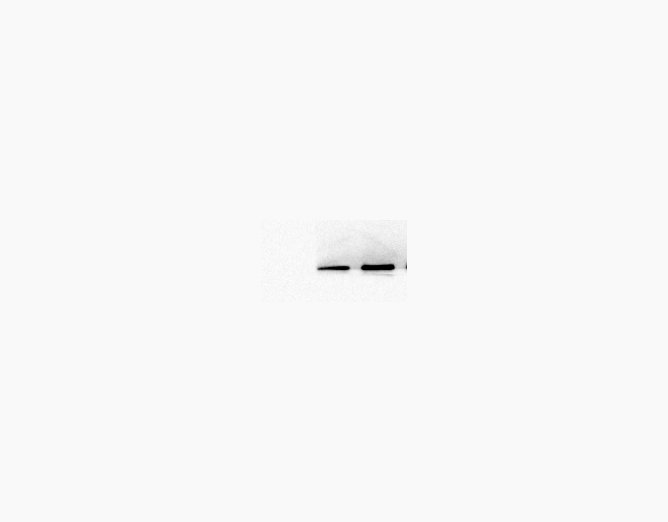

Supplement: Figure 4—source data 2. [file elife-85659-fig4-data2.zip › revised Figure 4 -raw unedited unlabelled versions of gel images with TIFF format/Source data 3 (Fig.4g)/IKKα.tif]

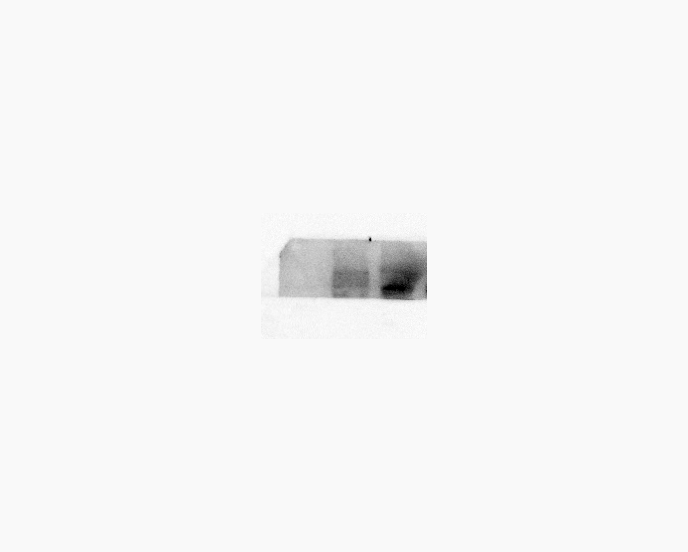

Supplement: Figure 4—source data 2. [file elife-85659-fig4-data2.zip › revised Figure 4 -raw unedited unlabelled versions of gel images with TIFF format/Source data 3 (Fig.4g)/P-IKKα β.tif]

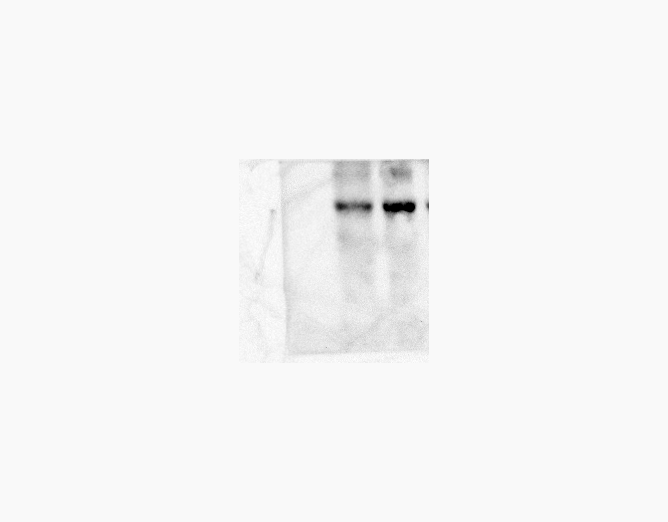

Supplement: Figure 4—source data 2. [file elife-85659-fig4-data2.zip › revised Figure 4 -raw unedited unlabelled versions of gel images with TIFF format/Source data 3 (Fig.4g)/p-IKBα.tif]

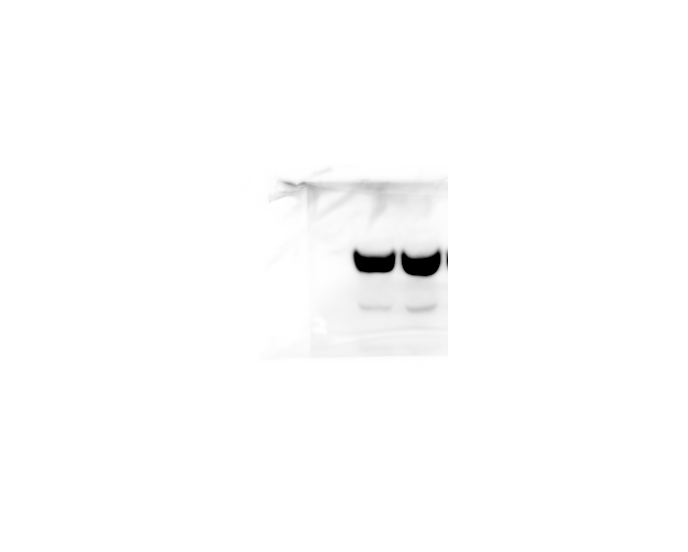

Supplement: Figure 4—source data 2. [file elife-85659-fig4-data2.zip › revised Figure 4 -raw unedited unlabelled versions of gel images with TIFF format/Source data 4 (Fig.4i)/GAPDH.tif]

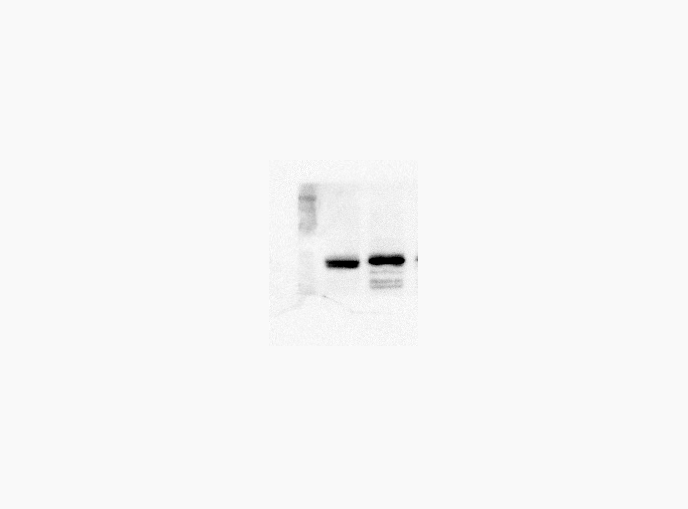

Supplement: Figure 4—source data 2. [file elife-85659-fig4-data2.zip › revised Figure 4 -raw unedited unlabelled versions of gel images with TIFF format/Source data 4 (Fig.4i)/Nuclear p65.tif]

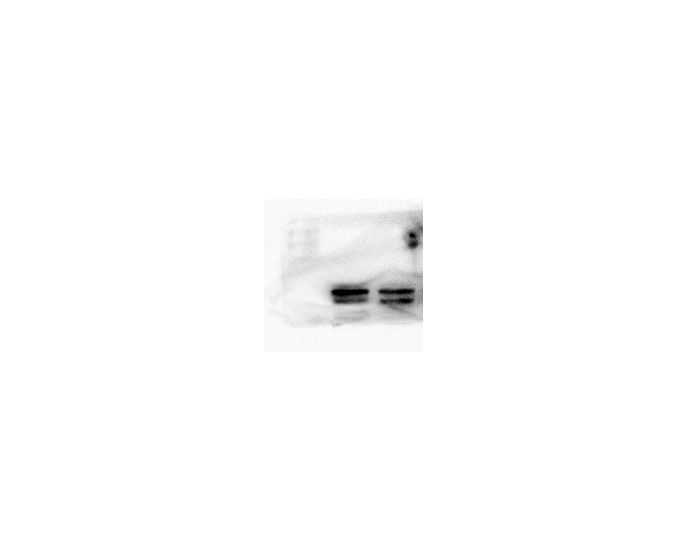

Supplement: Figure 4—source data 2. [file elife-85659-fig4-data2.zip › revised Figure 4 -raw unedited unlabelled versions of gel images with TIFF format/Source data 4 (Fig.4i)/cytoplasmic p65.tif]

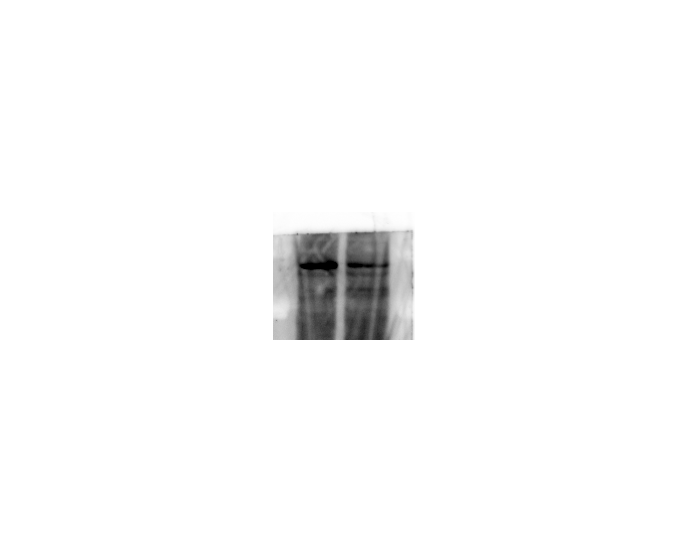

Supplement: Figure 4—source data 2. [file elife-85659-fig4-data2.zip › revised Figure 4 -raw unedited unlabelled versions of gel images with TIFF format/Source data 4 (Fig.4i)/lamin B.tif]

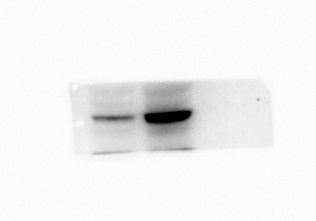

Supplement: Figure 4—figure supplement 1—source data 2. [file elife-85659-fig4-figsupp1-data2.zip › Figure 4-figure supplement 1-Raw western blot images with tiff format (Source data 1)/fam76b.tif]

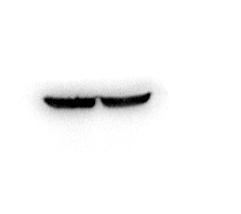

Supplement: Figure 4—figure supplement 1—source data 2. [file elife-85659-fig4-figsupp1-data2.zip › Figure 4-figure supplement 1-Raw western blot images with tiff format (Source data 1)/β-actin.tif]

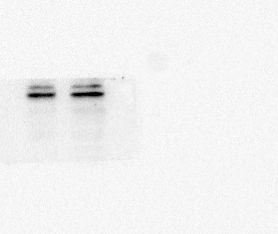

Supplement: Figure 4—figure supplement 2—source data 2. [file elife-85659-fig4-figsupp2-data2.zip › Figure 4-figure supplement 2-Raw western blot images with tiff format(Source data 1)/Figure 4-figure supplement 2-Raw western blot images with tiff format(Source data 1)/cytoplasmic hnRNPA2B1.tif]

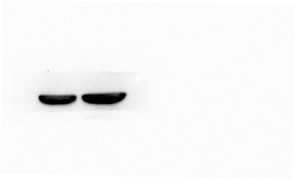

Supplement: Figure 4—figure supplement 2—source data 2. [file elife-85659-fig4-figsupp2-data2.zip › Figure 4-figure supplement 2-Raw western blot images with tiff format(Source data 1)/Figure 4-figure supplement 2-Raw western blot images with tiff format(Source data 1)/cytoplasmic β-actin.tif]

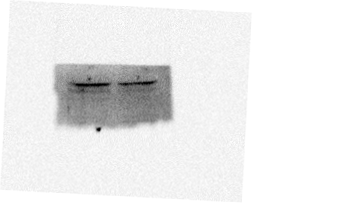

Supplement: Figure 4—figure supplement 2—source data 2. [file elife-85659-fig4-figsupp2-data2.zip › Figure 4-figure supplement 2-Raw western blot images with tiff format(Source data 1)/Figure 4-figure supplement 2-Raw western blot images with tiff format(Source data 1)/nuclear LaminB.tif]

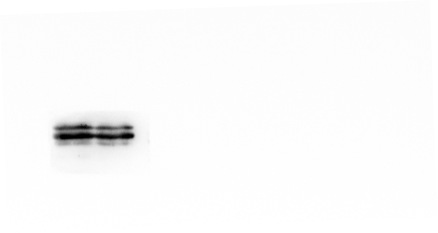

Supplement: Figure 4—figure supplement 2—source data 2. [file elife-85659-fig4-figsupp2-data2.zip › Figure 4-figure supplement 2-Raw western blot images with tiff format(Source data 1)/Figure 4-figure supplement 2-Raw western blot images with tiff format(Source data 1)/nuclear hnRNPA2B1.tif]

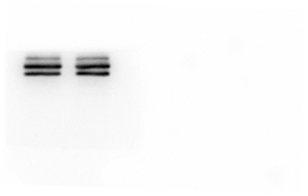

Supplement: Figure 4—figure supplement 2—source data 2. [file elife-85659-fig4-figsupp2-data2.zip › Figure 4-figure supplement 2-Raw western blot images with tiff format(Source data 1)/Figure 4-figure supplement 2-Raw western blot images with tiff format(Source data 1)/total hnRNPA2B1.tif]

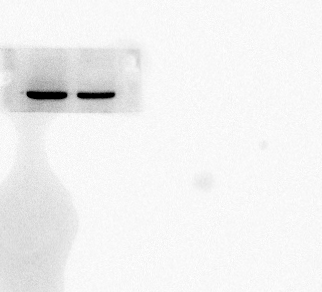

Supplement: Figure 4—figure supplement 2—source data 2. [file elife-85659-fig4-figsupp2-data2.zip › Figure 4-figure supplement 2-Raw western blot images with tiff format(Source data 1)/Figure 4-figure supplement 2-Raw western blot images with tiff format(Source data 1)/total β-actin.tif]

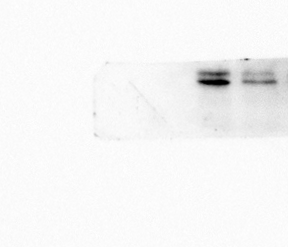

Supplement: Figure 4—figure supplement 3—source data 2. [file elife-85659-fig4-figsupp3-data2.zip › Figure 4-figure supplement 3-Raw western blot images with tiff format (Source data 1)/cytoplasmic hnRNPA2B1.tif]

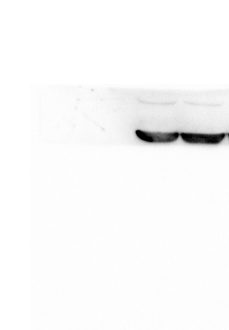

Supplement: Figure 4—figure supplement 3—source data 2. [file elife-85659-fig4-figsupp3-data2.zip › Figure 4-figure supplement 3-Raw western blot images with tiff format (Source data 1)/cytoplasmic β-actin.tif]

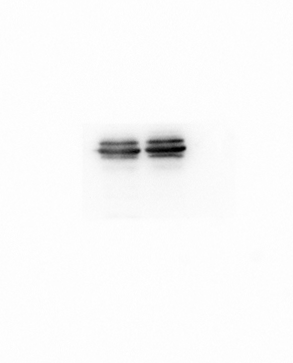

Supplement: Figure 4—figure supplement 3—source data 2. [file elife-85659-fig4-figsupp3-data2.zip › Figure 4-figure supplement 3-Raw western blot images with tiff format (Source data 1)/nuclear hnRNPA2B1.tif]

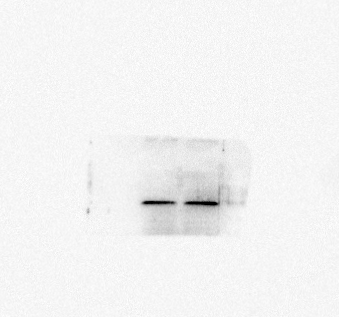

Supplement: Figure 4—figure supplement 3—source data 2. [file elife-85659-fig4-figsupp3-data2.zip › Figure 4-figure supplement 3-Raw western blot images with tiff format (Source data 1)/nuclear laminB.tif]

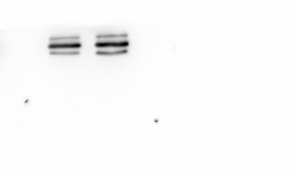

Supplement: Figure 4—figure supplement 3—source data 2. [file elife-85659-fig4-figsupp3-data2.zip › Figure 4-figure supplement 3-Raw western blot images with tiff format (Source data 1)/total hnRNPA2B1.tif]

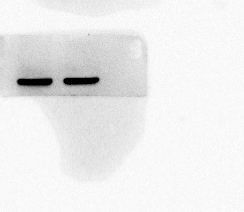

Supplement: Figure 4—figure supplement 3—source data 2. [file elife-85659-fig4-figsupp3-data2.zip › Figure 4-figure supplement 3-Raw western blot images with tiff format (Source data 1)/total β-actin.tif]

## Full unedited gel for Figure.5c

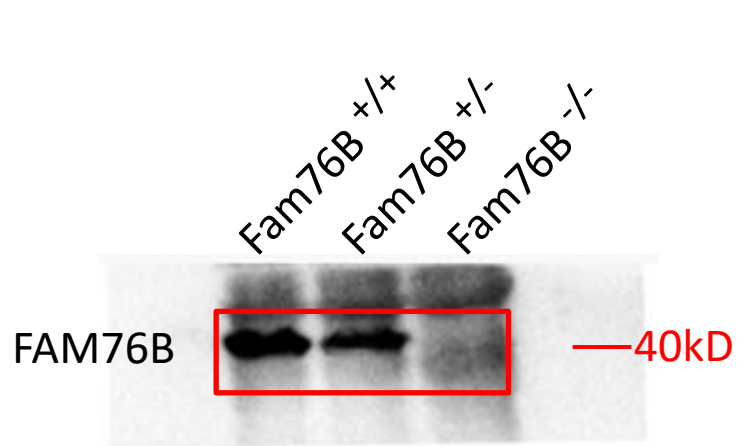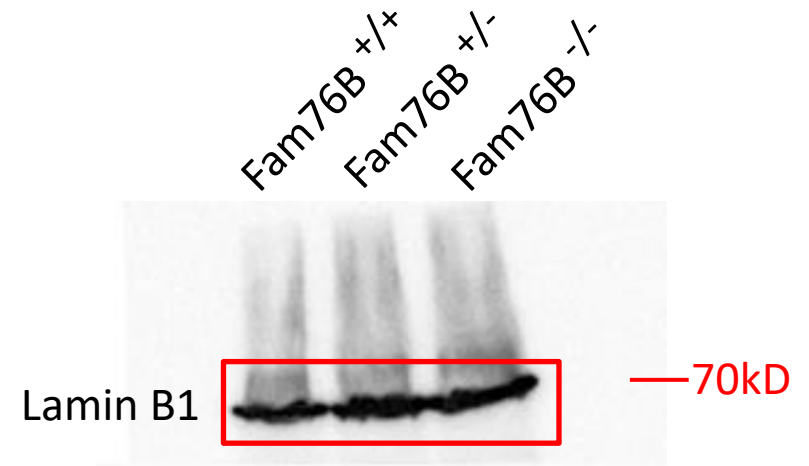

Supplement: Figure 5—source data 1. [file elife-85659-fig5-data1.pdf]

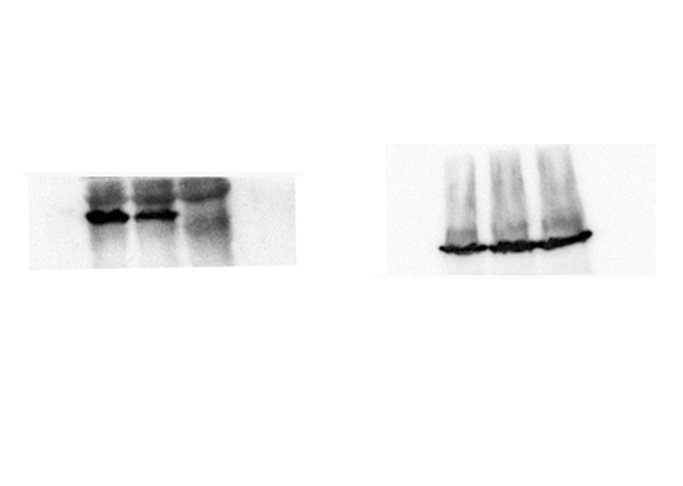

Supplement: Figure 5—source data 2. [file elife-85659-fig5-data2.zip › the raw unedited unlabelled versions of the gel images with tiff format ú¿Figure 5-source data 1)/Figure 5- source data 1.tif]
