# Supplementary material for: FAM76B regulates NF-κB-mediated inflammatory pathway by influencing the translocation of hnRNPA2B1
Source: eLife. 2023 Aug 10;12:e85659. doi: 10.7554/eLife.85659 (PMC10446823; doi:10.7554/eLife.85659)
Supplement: Figure 4—source data 1. [file elife-85659-fig4-data1.zip › Figure 4-Labeled uncropped western blot images (source data 1-4)/Figure 4-Source data 1.pdf]

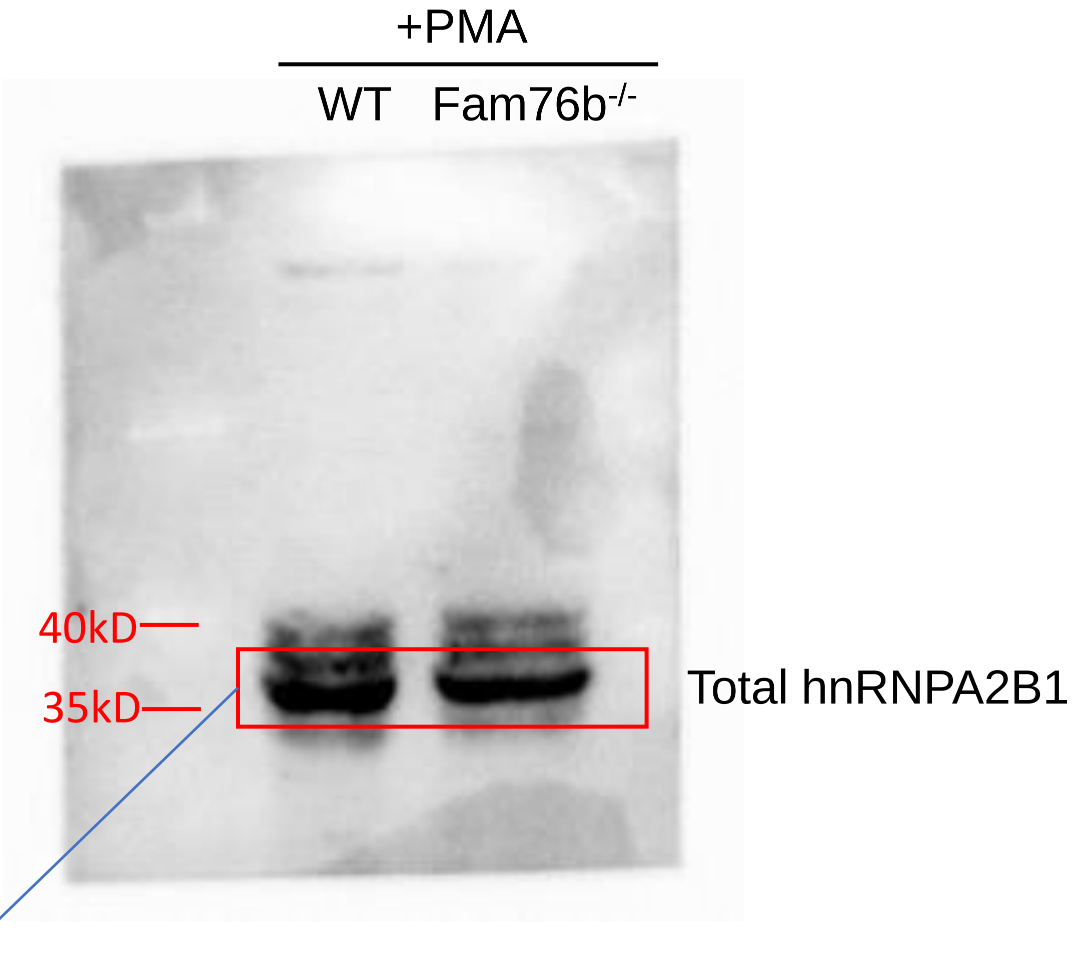

This lane corresponds to the band (Total hnRNPA2B1) of Figure 4b in the cropped images within the manuscript.

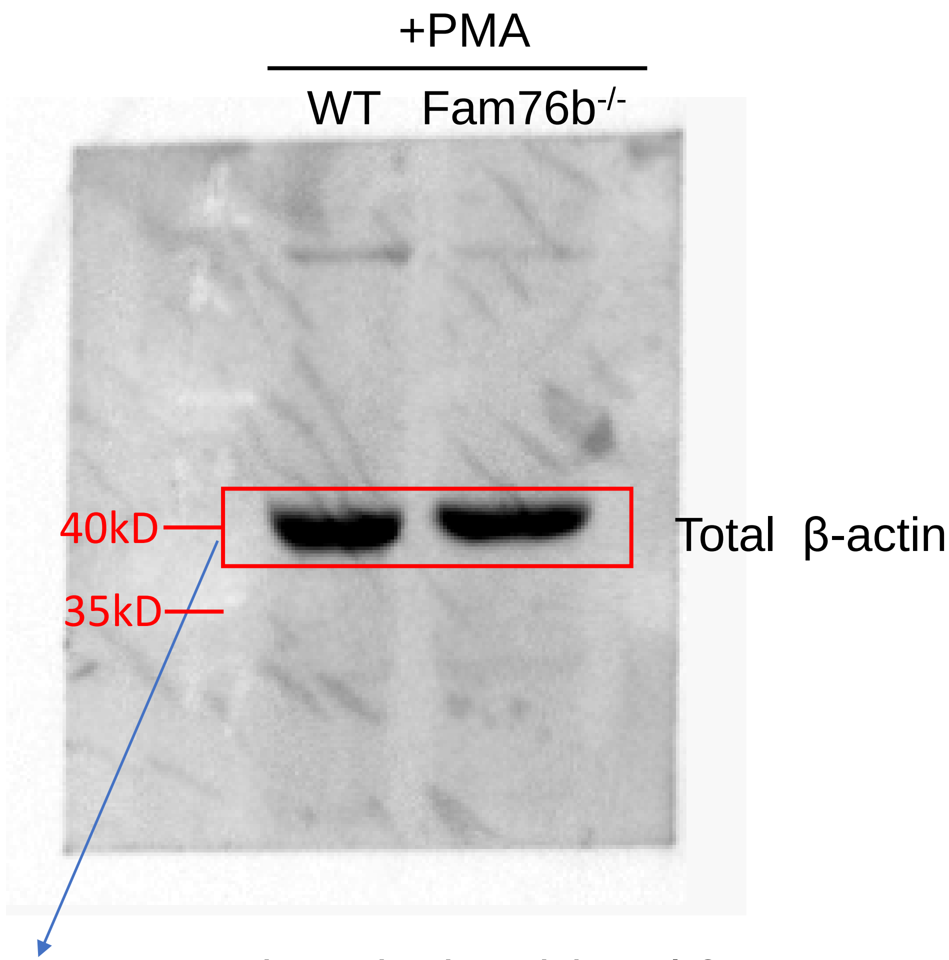

This lane corresponds to the band (Total β-actin) of Figure 4b in the cropped images within the manuscript.

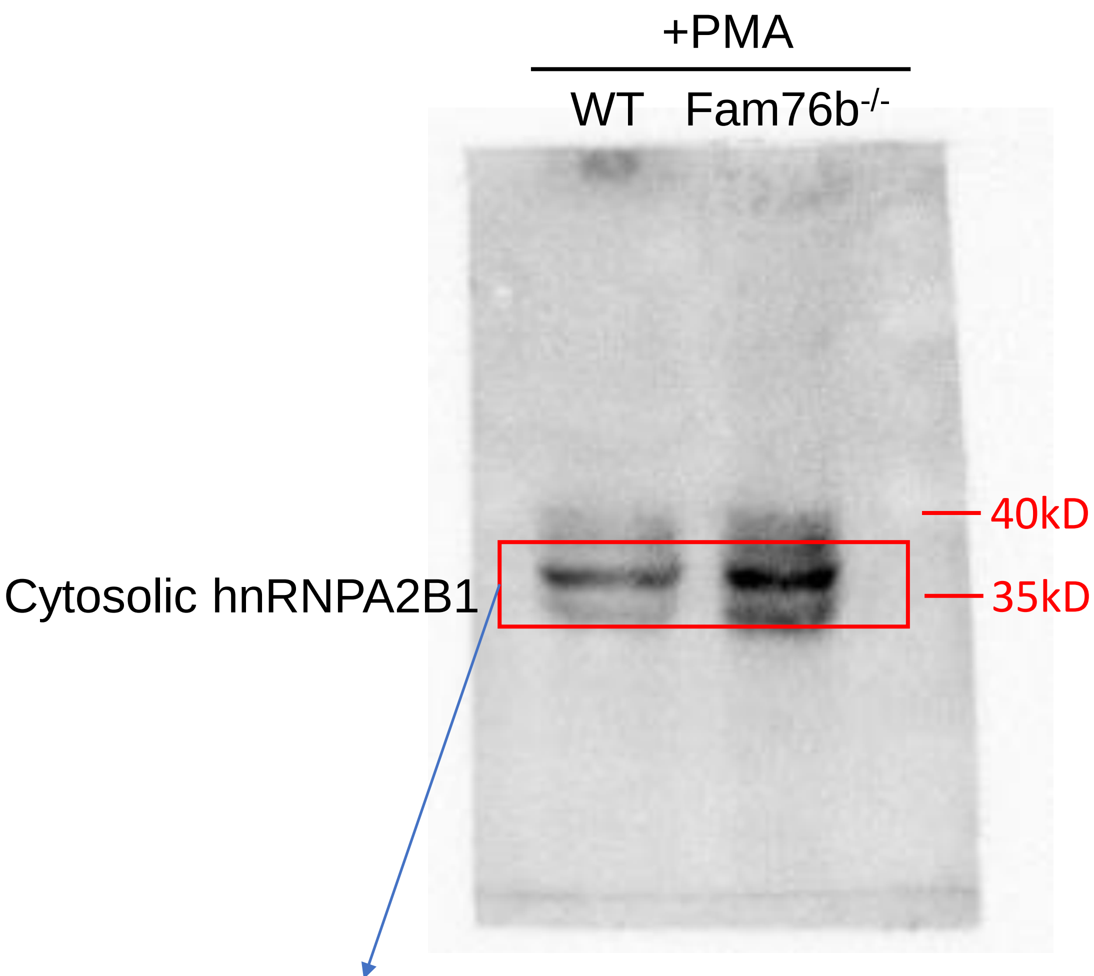

This lane corresponds to the band (Cytosolic hnRNPA2B1) of Figure 4b in the cropped images within the manuscript.

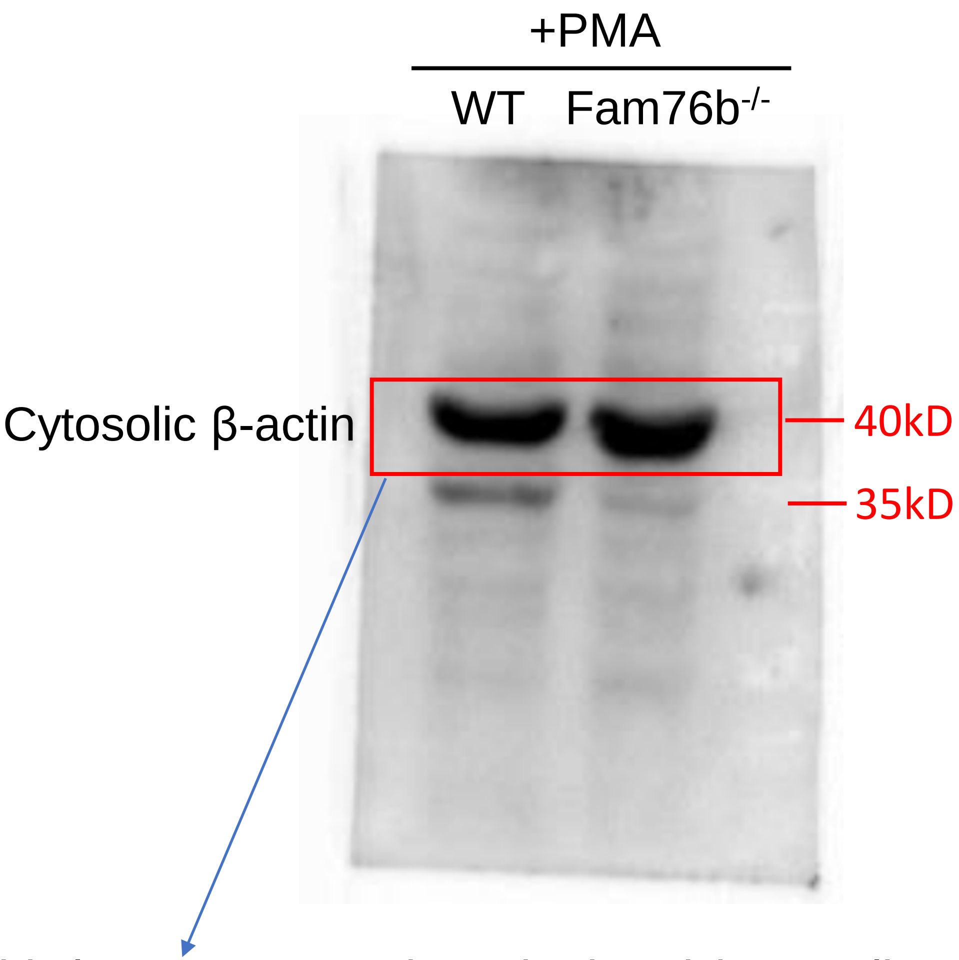

This lane corresponds to the band (Cytosolic β-actin) of Figure 4b in the cropped images within the manuscript.

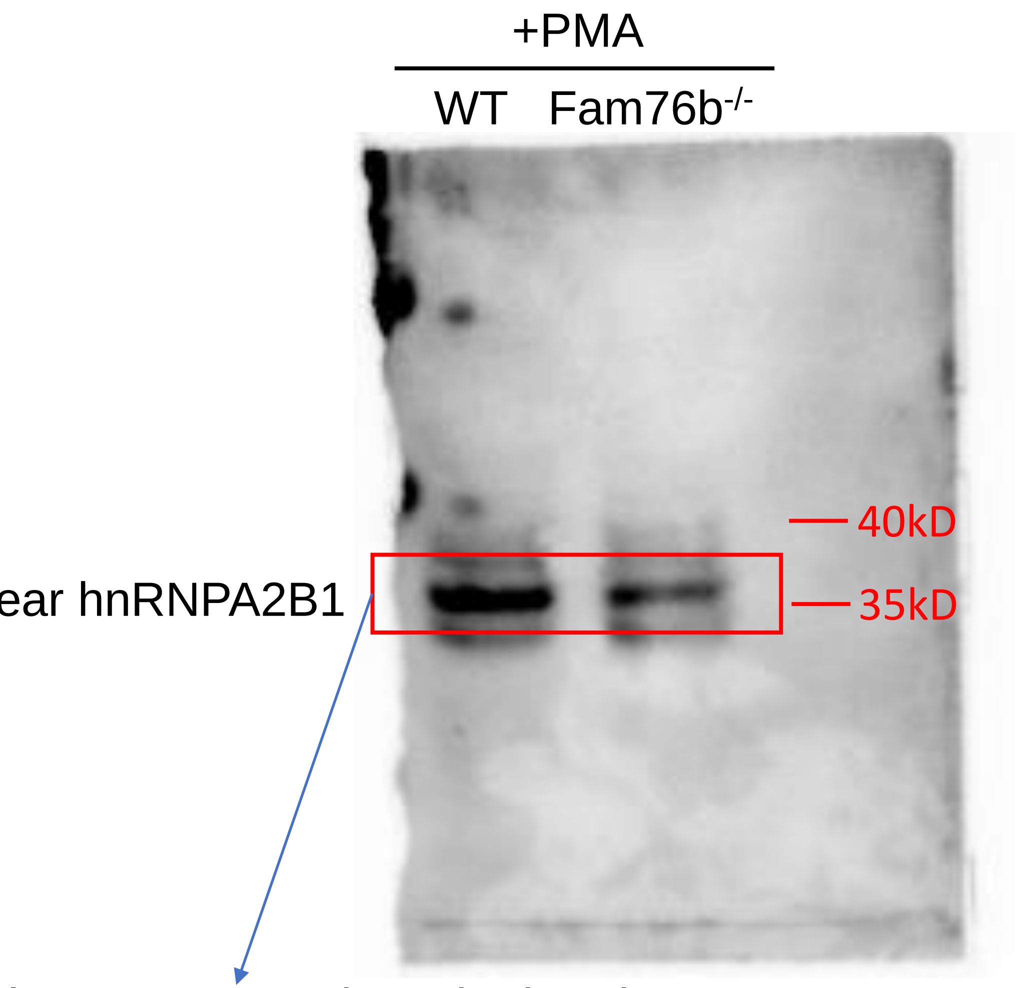

lane corresponds to the band
clear hnRNPA2B1) of Figure 4b in the
ped images within the manuscript.

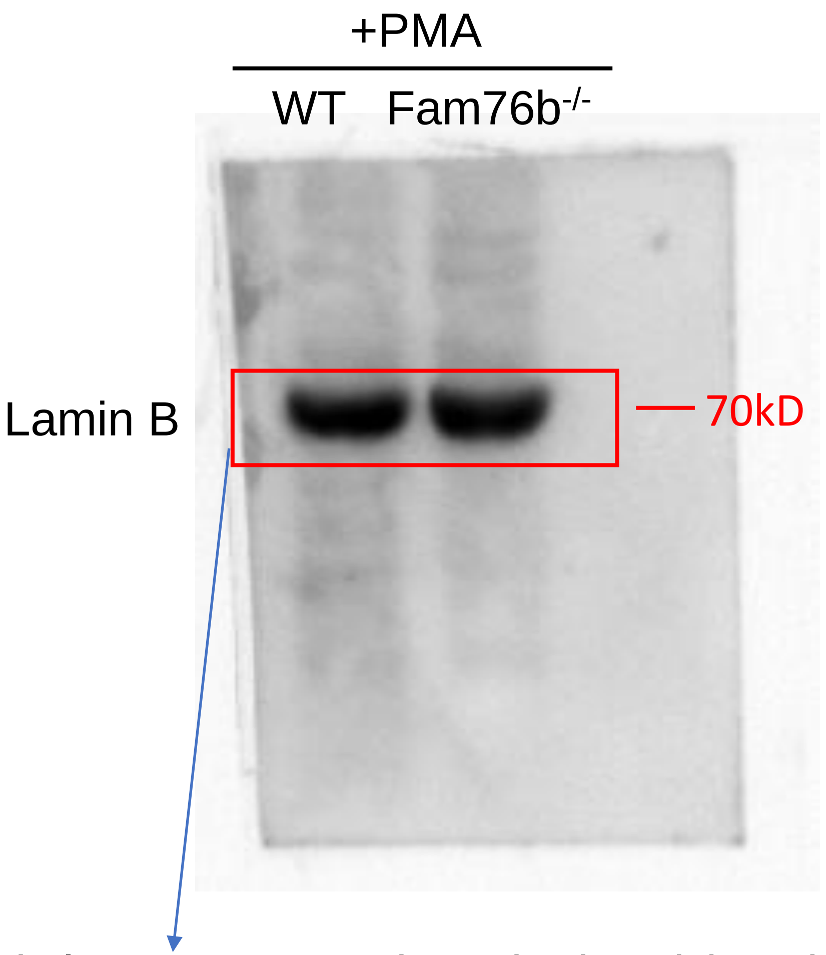

This lane corresponds to the band (Lamin B) of Figure 4b in the cropped images within the manuscript.
