# Supplementary material for: FAM76B regulates NF-κB-mediated inflammatory pathway by influencing the translocation of hnRNPA2B1
Source: eLife. 2023 Aug 10;12:e85659. doi: 10.7554/eLife.85659 (PMC10446823; doi:10.7554/eLife.85659)
Supplement: Figure 4—figure supplement 2—source data 1. [file elife-85659-fig4-figsupp2-data1.pdf]

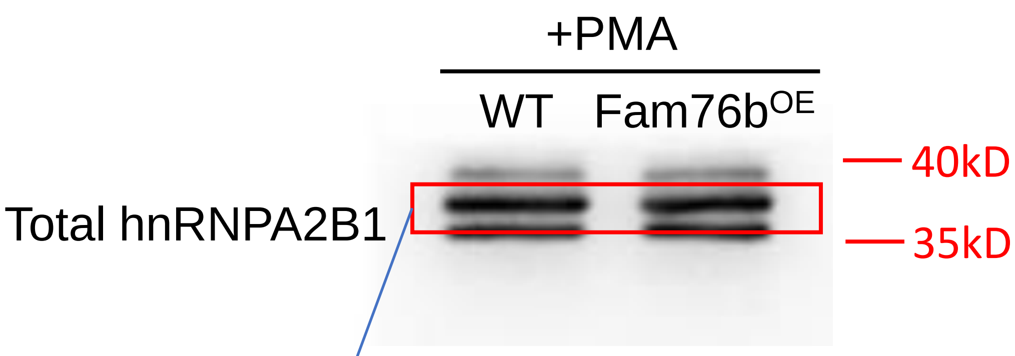

This lane corresponds to the band (Total hnRNPA2B1) of Figure 4-figure supplement 2b in the cropped images within the manuscript.

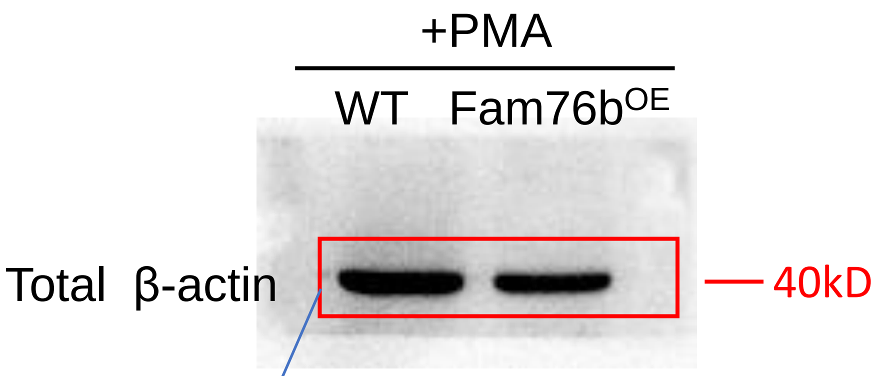

This lane corresponds to the band (Total β-actin) of Figure 4-figure supplement 2b in the cropped images within the manuscript.

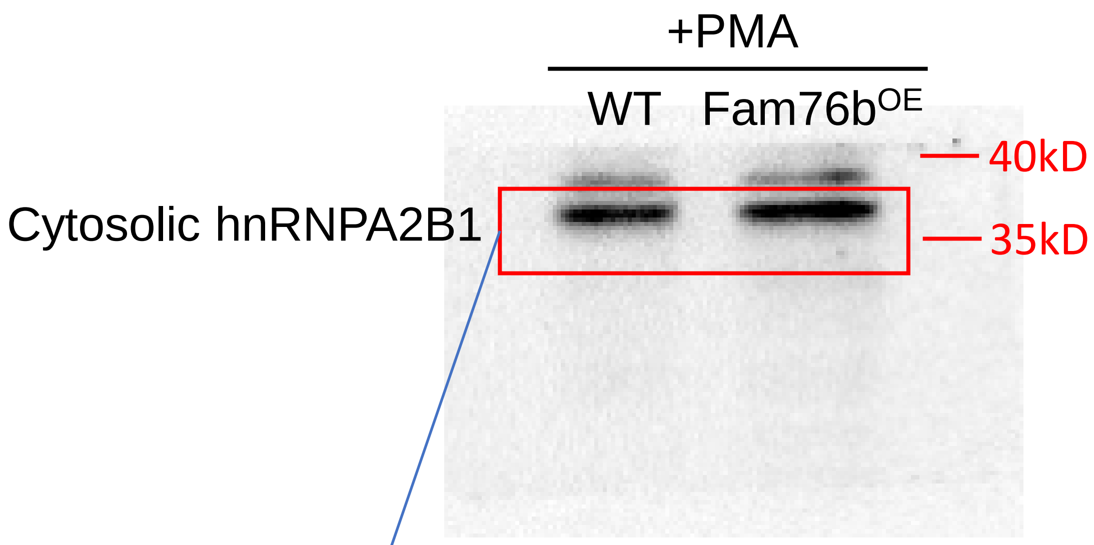

This lane corresponds to the band (Cytosolic hnRNPA2B1) of Figure 4-figure supplement 2b in the cropped images within the manuscript.

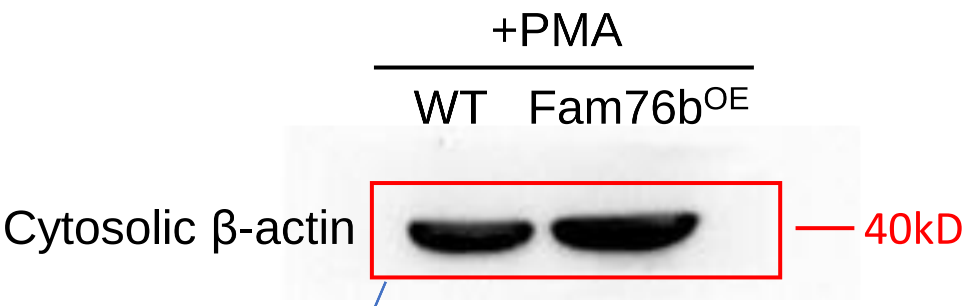

This lane corresponds to the band (Cytosolic β-actin) of Figure 4-figure supplement 2b in the cropped images within the manuscript.

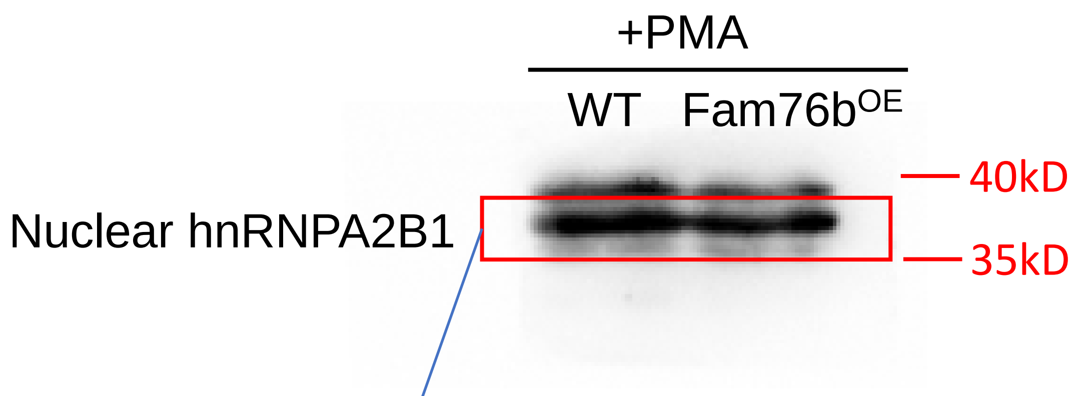

This lane corresponds to the band (Nuclear hnRNPA2B1) of Figure 4-figure supplement 2b in the cropped images within the manuscript.

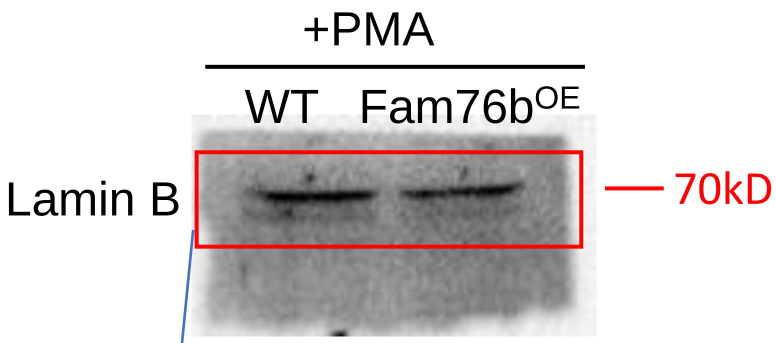

This lane corresponds to the band (Lamin B) of Figure 4-figure supplement 2b in the cropped images within the manuscript.
