# Supplementary material for: FAM76B regulates NF-κB-mediated inflammatory pathway by influencing the translocation of hnRNPA2B1
Source: eLife. 2023 Aug 10;12:e85659. doi: 10.7554/eLife.85659 (PMC10446823; doi:10.7554/eLife.85659)
Supplement: Supplementary file 1. [file elife-85659-supp1.docx]

**Supplementary File 1. Proteins that interact with FAM76B and their interacting scores and ranks**

| Protein IDs | Protein names | Gene names | Score | Rank |
| --- | --- | --- | --- | --- |
| P22626 | Heterogeneous nuclear ribonucleoproteins A2/B1 | HNRNPA2B1 | 164.67 | 5 |
| P07355 | Annexin A2 | ANXA2 | 60.366 | 11 |
| P46776 | 60S ribosomal protein L27a | RPL27A | 31.429 | 24 |
| Q8WWM7 | Ataxin-2-like protein. This gene encodes an ataxin type 2-related protein of unknown function. | ATXN2L | 30.629 | 25 |
| P09651; Q32P51 | Heterogeneous nuclear ribonucleoprotein A1; Heterogeneous nuclear ribonucleoprotein A1-like 2 | HNRNPA1; HNRNPA1L2 | 27.543 | 28 |
| P51991 | Heterogeneous nuclear ribonucleoprotein A3 | HNRNPA3 | 27.536 | 29 |
| P55072 | Transitional endoplasmic reticulum ATPase | VCP | 10.17 | 56 |
| O75369; P21333; Q14315 | Filamin-B; Filamin-A; Filamin-C | FLNB; FLNA; FLNC | 5.9136 | 99 |
| P35637; Q92804 | RNA-binding protein FUS | FUS; TAF15 | 5.866 | 101 |
| P45974 | Ubiquitin carboxyl-terminal hydrolase 5 | USP5 | 5.6403 | 133 |
