# Supplementary material for: FAM76B regulates NF-κB-mediated inflammatory pathway by influencing the translocation of hnRNPA2B1
Source: eLife. 2023 Aug 10;12:e85659. doi: 10.7554/eLife.85659 (PMC10446823; doi:10.7554/eLife.85659)
Supplement: Supplementary file 2. [file elife-85659-supp2.docx]

**Supplementary File 2. Primers used for real-time PCR**

| Gene | Sequence | |
| --- | --- | --- |
| Human *GAPDH* | Forward | 5’-GCACCGTCAAGGCTGAGAAC-3’ |
|  | Reverse | 5’-TGGTGAAGACGCCAGTGGA-3’ |
| Human *IL6* | Forward | 5’-GGATTCAATGAGGAGACTTGCC-3’ |
|  | Reverse | 5’-TGGCATTTGTGGTTGGGTCA-3’ |
| Human *PTGS2* | Forward | 5’-CAAATTGCTGGCAGGGTTGC-3’ |
|  | Reverse | 5’-AGGGCTTCAGCATAAAGCGT-3’ |
| Human *TNFα* | Forward | 5’-TCCCCAGGGACCTCTCTCTA-3’ |
|  | Reverse | 5’-GAGGGTTTGCTACAACATGGG-3’ |
| Human *IL10* | Forward | 5’-TACGGCGCTGTCATCGATTT-3’ |
|  | Reverse | 5’-AAGGTTTCTCAAGGGGCTGG-3’ |
| Human *FAM76B* | Forward | 5’-AGCAGATAGTGGGGGAACAGACAA-3’ |
|  | Reverse | 5’-TTTTGGCCTGAAGTTGTTCCACAGT-3’ |
| Human *hnRNPA2B1* | Forward | 5’-CAGTTCTCACTACAGCGCCA-3’ |
|  | Reverse | 5’-GCTCTGGTGTCTTCTGCCAT-3’ |
| Mouse *GAPDH* | Forward | 5’-AAGGCCGGGGCCCACTTGAA-3’ |
|  | Reverse | 5’-AGCAGTTGGTGGTGCAGGATGC-3’ |
| Mouse *FAM76B* | Forward | 5’-ACTGTGGAACAACTCCAGGCCAA-3’ |
|  | Reverse | 5’-CAGGGAGATGTTAGCACGCTTCCA-3’ |
| Mouse *IL6* | Forward | 5’-CCTTCCTACCCCAATTTCCAAT-3’ |
|  | Reverse | 5’-GCCACTCCTTCTGTGACTCCAG-3’ |
| Mouse *TNFα* | Forward | 5’-GGCAGGTCTACTTTGGAGTC-3’ |
|  | Reverse | 5’-TCGAGGCTCCAGTGAATTCG-3’ |
