# Supplementary material for: FAM76B regulates NF-κB-mediated inflammatory pathway by influencing the translocation of hnRNPA2B1
Source: eLife. 2023 Aug 10;12:e85659. doi: 10.7554/eLife.85659 (PMC10446823; doi:10.7554/eLife.85659)
Supplement: Supplementary file 3. [file elife-85659-supp3.docx]

**Supplementary File 3.** **Other primers used in the study**

| Gene | Sequence | |
| --- | --- | --- |
| *FAM76B* sgRNA target genome region | Forward | 5’-CTTGTGTAACCCACCCTTTGT-3’ |
|  | Reverse | 5’-TTGTTGCAGAGGATTTATGGCT-3’ |
| *IL6* promoter | Forward | 5’-AGGTACCTTCCTATTTAAAAAACAC-3’ |
|  | Reverse | 5’-TAAGCTTAGCTGGGCTCCTGGAGGG-3’ |
| Human *P50* | Forward | 5’-CCCTCGAGATGGCAGAAGATGATCCATA-3’ |
|  | Reverse | 5’-CCTCTAGAAACTTTCCCAAAGAGGTTTA-3’ |
| Human *P65* | Forward | 5’-CCCCCTCGAGATGGGACGAACTGTTCCC  CCT-3’ |
|  | Reverse | 5’-CCTCTAGAGGAGCTGATCTGACTCAGCA-3’ |
| Mouse *FAM76B* genotyping | Forward | 5’-GCAGAGATTGGGTGCAGACT-3’ |
|  | Reverse | 5’-GAGACCCAATCTCACTCTTTG-3’ |
|  | V76 Reverse | 5’-CCAATAAACCCTCTTGCAGTTGC-3’ |
|  |  |  |
| *hnRNPA2B1 RRM1 NLS* | Forward | 5’-ACTCGAGATGGAGAAAACTTTAGAAAC-3’ |
| *hnRNPA2B1 RRM2 NLS*  *hnRNPA2B1 RGD NLS* | Reverse  Forward  Reverse  Forward  Reverse | 5’- TTCTAGACACCTTCCTCTTCTTCTTGGGAGTT  ACATGAGCCCCTGGTT-3’  5’-ACTCGAGATGGTGAAGAAGCTGTTTGTTGG-3’  5’- TACTAGTCACCTTCCTCTTCTTCTTGGGCCTAG  AACTCTGAACTTCCT-3’  5’-ACTCGAGATGAGTGGAAGAGGAGGCAACT  T-3’  5’-TTCTAGACACCTTCCTCTTCTTCTTGGGGTATC  GGCTCCTCCCACC -3’ |
