# Supplementary material for: FAM76B regulates NF-κB-mediated inflammatory pathway by influencing the translocation of hnRNPA2B1
Source: eLife. 2023 Aug 10;12:e85659. doi: 10.7554/eLife.85659 (PMC10446823; doi:10.7554/eLife.85659)
Supplement: Supplementary file 4. [file elife-85659-supp4.docx]

**Supplementary File 4. Antibodies used in the study**

| Antibody | Manufacturer | | Catalogue number | |
| --- | --- | --- | --- | --- |
| FITC anti-mouse CD3 | | BioLegend | | 100203 |
| FITC Rat IgG2b, κ Isotype Ctrl | | BioLegend | | 400605 |
| PerCP/Cy5.5 anti-mouse/human CD11b | | BioLegend | | 101227 |
| PerCP/Cy5.5 Rat IgG2b, κ Isotype Ctrl | | BioLegend | | 400631 |
| APC anti-mouse CD19 | | BioLegend | | 152409 |
| APC Rat IgG2a, κ Isotype Ctrl Antibody | | BioLegend | | 400511 |
| TruStain fcX™ (anti-mouse CD16/32) | | BioLegend | | 101319 |
| FAM76B monoclonal antibody (mouse) | | Homemade | |  |
| Rabbit anti-hnRNPA2B1 polyclonal antibody | | Abcam | | ab31645 |
| Mouse anti-Flag monoclonal antibody | | Cell signaling | | 14793 |
| Goat anti-IBA1 polyclonal antibody | | Abcam | | ab5076 |
| Mouse anti-GAPDH monoclonal antibody | | Proteintech | | 60004-1-Ig |
