# Supplementary material for: FAM76B regulates NF-κB-mediated inflammatory pathway by influencing the translocation of hnRNPA2B1
Source: eLife. 2023 Aug 10;12:e85659. doi: 10.7554/eLife.85659 (PMC10446823; doi:10.7554/eLife.85659)
Supplement: Supplementary file 5. [file elife-85659-supp5.docx]

**Supplementary File 5. Patient demographics**

| Neuropathologic diagnosis | Number  of cases | Sex  M/F | Age at death  (mean ± SD) |
| --- | --- | --- | --- |
| CON | 6 | 3/3 | 77.8±7.8 |
| AD | 5 | 2/3 | 77.3±8.4 |
| FTLD-tau | 6 | 2/4 | 72.3±3.2 |
| FTLD-TDP | 6 | 4/2 | 71.5±6.9 |

CON, normal control; AD, Alzheimer’s disease; FTLD-tau, frontotemporal lobar degeneration with tau pathology; FLTD-TDP, frontotemporal lobar degeneration with TAR DNA-binding protein 43 inclusions.
